# Ozone therapy as an adjuvant for endondontic protocols: microbiological – *ex vivo* study and citotoxicity analyses

**DOI:** 10.1590/1678-775720160029

**Published:** 2016

**Authors:** Carlos Goes NOGALES, Marina Beloti FERREIRA, Antonio Fernando MONTEMOR, Maria Filomena de Andrade RODRIGUES, José Luiz Lage-MARQUES, João Humberto ANTONIAZZI

**Affiliations:** 1 - Universidade de São Paulo, Faculdade de Odontologia, Departamento de Endodontia, São Paulo, SP, Brasil.; 2 - Instituto de Pesquisas Tecnológicas do Estado de São Paulo - IBL Núcleo de Bionanomanufatura, São Paulo, SP, Brasil.; 3 - Instituto de Pesquisas Tecnológicas do Estado de São Paulo, Departamento de Biotecnologia , São Paulo, SP, Brasil.

**Keywords:** Ozonated water, Ozone, Endodontic, Cytotoxicity, Antimicrobial activity

## Abstract

**Objectives:**

This study evaluated the antimicrobial efficacy of ozone therapy in teeth contaminated with *Pseudomonas aeruginosa*, *Enterococcus faecalis*, and *Staphylococcus aureus* using a mono-species biofilm model. Parallel to this, the study aimed to evaluate the cytotoxicity of ozone for human gingival fibroblasts. Material and Methods: One hundred and eighty single-root teeth were contaminated with a mono-species biofilm of *Enterococcus faecalis*, *Pseudomonas aeruginosa*, and *Staphylococcus aureus*. Groups were formed: Group I – control; Group II – standard protocol; Group III – standard protocol + ozone gas at 40 µg/mL; and Group IV – standard protocol + aqueous ozone at 8 µg/mL. In parallel, human gingival fibroblasts were submitted to the MTT test. Cells were plated, then ozone was applied as follows: Group I (control) – broth medium; Group II – aqueous ozone at 2 µg/mL; Group III – aqueous ozone at 5 µg/mL; and Group IV – aqueous ozone at 8 µg/mL. Data were submitted to the Kruskal Wallis test and Bonferroni *post hoc* analyses to assess microbiology and cytotoxicity, respectively (p<0.05%).

**Results:**

The results revealed antimicrobial efficacy by Group IV with no CFU count. The cytotoxicity assay showed Groups III and IV to be the most aggressive, providing a decrease in cell viability at hour 0 from 100% to 77.3% and 68.6%, respectively. Such a decrease in cell viability was reverted, and after 72 hours Groups III and IV provided the greatest increase in cell viability, being statistically different from Groups I and II.

**Conclusion:**

According to the applied methodology and the limitations of this study, it was possible to conclude that ozone therapy improved the decontamination of the root canal *ex vivo*. Ozone was toxic to the cells on first contact, but cell viability was recovered. Thus, these findings suggest that ozone might be useful to improve root canal results.

## INTRODUCTION

In Endodontic therapy, success is closely linked to the removal of debris and the elimination of remaining microorganisms and byproducts. To fulfill this requirement, the clinician must use Endodontic instruments associated with chemical substances. In order to improve the decontamination procedures due to the inaccessible areas in the root canal system, more effective antimicrobial treatment strategies and substances should be studied^22^.

Ozone is both antimicrobial^2^ ^,^ ^6^ ^,^ ^7^ ^,^ ^10^ ^,^ ^12^ ^-^ ^18^ and biocompatible^8^. Studies have shown the important efficacy of aqueous ozone^18^ especially when associated with ultrasonic activation^10^ ^,^ ^17^. The efficacy is statistically comparable to the highest concentrations of sodium hypochlorite^10^ ^,^ ^14^.

Ozone is an extremely oxidant agent that reacts directly with the fatty-acids of the microorganisms’ cell walls. In parallel, ozone increases the production of adenosine triphosphate (ATP) by mitochondria, which leads to a metabolic improvement and to healing of inflammatory/infectious processes^1^ ^,^ ^2^ ^,^ ^4^ ^,^ ^8^ ^,^ ^11^ ^,^ ^15^ ^,^ ^17^ ^,^ ^19^.

Ozone therapy is proposed as a coadjuvant to Endodontic treatment in order to improve the decontamination process^6^ ^,^ ^7^ ^,^ ^10^ ^,^ ^12^ ^-^ ^17^. Furthermore due to the low level of hazardousness^11^ ^,^ ^17^ and the high level of biocompatibility^8^, it might directly affect the healing process. Studies have proven its ability to interact effectively with microbiota in the root canal system and therefore to eliminate microorganisms^1^ ^,^ ^3^ ^,^ ^5^ ^-^ ^7^ ^,^ ^10^ ^,^ ^12^ ^-^ ^17^. This feature is so remarkable that bacterial resistance has not been reported in the scientific literature.

Thus, the aim of this study was to evaluate the antimicrobial efficacy of ozone therapy in teeth contaminated with *Pseudomonas aeruginosa, Enterococcus faecalis*, and *Staphylococcus aureus* using a mono-species biofilm model. In parallel, this study also aimed to evaluate the cytotoxicity of ozone to human gingival fibroblasts.

## MATERIAL AND METHODS

This study was approved by the Ethics Committee of the Dental School of the University of São Paulo (154/06).

### Ozone standardization

Ozone standardization procedures followed the protocol described in a prior study^2^. An ozone generator (Philozon, Balneario Camboriú, SC, Brazil) was employed in this study. It has a self-calibration system that provides ozone at a flux rate of 1 L/min. Ozone was generated by electrical discharge on pure oxygen (Respirox LTDA, Sao Paulo, SP, Brazil).

A glass flask was attached to the ozone generator, and ozone was bubbled into the cold bi-distilled water for 5 minutes. Then the solution was collected from the glass flask and applied to the experimental group. The final concentration of aqueous ozone was confirmed by a colorimetric kit (CHEMets™ Kit Ozone K-7402, CheMets, Midland, Washington, USA).

### Preparation of contaminated specimens

*Enterococcus faecalis* (ATCC 29212), *Pseudomonas aeruginosa* (ATCC 27853), and *Staphylococcus aureus* (ATCC 6538) were cultivated in a mono-species biofilm in Tryptone Soya Broth (TSB - Oxoid, Cambridge, London, UK) at 37°C for 24 hours in an aerobic chamber. The bacterium concentrations were spectrophotometrically adjusted.

One hundred and eighty extracted teeth were selected. *Enterococcus faecalis*, *Pseudomonas aeruginosa*, and *Staphylococcus aureus* were grown in a mono-species biofilm and each micro organism was inoculated in 60 teeth. The tooth selection was based on the following inclusion criteria: complete rizogenesis, single root, and single canal. All specimens underwent radiographic testing to verify these criteria. The external surfaces were cleaned with a Gracey curette, and all specimens were kept in saline solution until usage. In order to standardize the intracanal volume, crowns were removed with carborundum discs providing the root length of 17 mm. The apical foramen was unblocked with a #10 file. Serial instrumentation was performed until reaching K-file #25 (Dentsply-Maillefer, Ballaigues, Swiss). Irrigation with 3 mL of 1% sodium hypochlorite was performed after each instrument was used. Gates-Glidden burs (Dentsply-Maillefer, Ballaigues, Swiss) #2 and #3 were applied to the canal entrance. Then, the root canals were filled with 17% EDTA for 3 minutes, and then with 5 mL of physiologic saline solution. The apical region was sealed with a light-cured resin composite (Z250, 3M Dental Products, St Paul, MN, USA), and the outer root was covered with ethyl-cyanoacrylate (Henkel, Itapevi, SP, Brazil). All of the teeth were sterilized for 15 minutes.

The specimens were randomly added to a polyethylene plate and fastened with acrylic resin. These plaques were separated according to the microorganism. Then, 10 µL of each microbial suspension was inoculated into the root canals and incubated for 7 days. The broth was renewed every 48 hours. After incubation, the teeth were then divided into the experimental groups:

Group I (45 teeth) – contamination control: The teeth were immersed individually in 10 mL of 0.1% sodium thiosulfate solution and Vortex. Serial dilution was performed, followed by plating and aerobically incubating for 24 hours at 37°C. Then, CFUs were counted.

Group II (45 teeth) – treatment control: The teeth were mechanically prepared using 25/.06, 30/.06, 35/.06, and 40/.06 EndoSequence files (Brasseler USA^®^ Dental, Savannah, GA, USA) in a Rotary Master rotary device (J. Morita MFG. Corp, Suita City, Osaka, Japan) calibrated to 700 rpm and 3 N torque. The teeth were chemically prepared with 1% sodium hypochlorite associated with Endo-PTC gel (urea peroxide, carbopol, polyethylene glycol, and Tween 80). Each instrument change was followed by a 2 mL rinse with 1% sodium hypochlorite. At the end of the chemo/mechanical procedures, the teeth were submitted to a 10 mL intracanal rinse of 17% EDTA and a final irrigation of 1% sodium hypochlorite, comprising a total volume of 15 mL of 1% sodium hypochlorite. Following this, the teeth were submitted to the same procedures as described in Group I for CFU counting.

Group III (45 teeth): The teeth were mechanically prepared using 25/.06, 30/.06, 35/.06, and 40/.06 EndoSequence files (Brasseler USA^®^ Dental, Savannah, GA, USA) in a Rotary Master rotary device (J. Morita MFG. Corp, Suita City, Osaka, Japan) calibrated to 700 rpm and 3 N torque. The teeth were chemically prepared with 1% sodium hypochlorite associated with Endo-PTC gel (urea peroxide, carbopol, polyethylene glycol, and Tween 80). Each instrument change was followed by a 2 mL rinse with 1% sodium hypochlorite. At the end of the chemo/mechanical procedures, the teeth were submitted to 10 mL intracanal rinse of 17% EDTA and a final irrigation of 1% sodium hypochlorite, comprising a total volume of 15 mL of 1% sodium hypochlorite. The root canal was dried with paper points and a 10 mL syringe (Terumo, Shibuya-ku, Tokyo, Japan) was connected to the ozone generator, filled with ozone gas in a concentration of 40 µg/mL and injected into the root canal with NaviTips^®^ (Ultradent Products Inc, South Jordan, UT, USA) for 30 seconds. Following this, the teeth were submitted to the same procedures described in Group I for CFU count.

Group IV (45 teeth): The teeth were mechanically prepared using 25/.06, 30/.06, 35/.06, and 40/.06 EndoSequence files (Brasseler, Savannah, GA, USA). The teeth were chemically prepared with 1% sodium hypochlorite associated with Endo-PTC gel (urea peroxide, carbopol, polyethylene glycol, and Tween 80). Each instrument change was followed by a 2 mL rinse with 1% sodium hypochlorite. At the end of the chemo/mechanical procedures, the teeth were submitted to 10 mL of intracanal rinse of 17% EDTA and a final irrigation of 1% sodium hypochlorite. Immediately after the final irrigation with 1% sodium hypochlorite, the root canal was dried and 10 mL of aqueous ozone at 8 µg/mL, preparared as decribed above, was applied with an irrigation point (NaviTips^®^, Ultradent Products Inc, South Jordan, UT, USA), in a forward and backward movement in order to provide an intracanal turbulence. Following this, the teeth were submitted to the same procedures described in Group I for CFU count.

After 24 hours of aerobic incubation, the CFUs were counted and the data was submitted to statistical analysis. First, the data was submitted to a Kolmogorov-Smirnov test and then a Levene test to evaluate their normality and homogeneity, respectively. Considering the data related to the microbiological counts, for all bacteria species, the data did not follow a normal or homogeneous distribution. Therefore, the data was presented as a median and an interquartile range, and comparisons among the four groups were carried out through Kruskal-Wallis tests.

### Cytotoxicity assay in fibroblast cell

A human gingival fibroblast cell-line (FMM1), provided by the Basic Research Laboratory at the Restorative Department of Dental School of the University of São Paulo, was employed.

All procedures were performed in a laminar flux chamber under rigid protocols of disinfection and room temperature. The entire experiment was performed in triplicate.

A cryogenic tube containing cells was defrosted. Afterwards, the suspended cells were transferred to a centrifuge tube containing 5 mL of DME broth (Dulbecco’s modified Eagle’s medium; Sigma Chemical Co., St Louis, MO, USA), 10% bovine fetal serum (Cultilab, Campinas, SP, Brazil), and 1% of antibiotic-antimycotic (Sigma Chemical Co., St Louis, MO, USA). The cell growth was monitored every 24 hours using an inverted phase microscope, and the broth was exchanged every two days, according to cell metabolism.

Cell viability analyses using direct contact with the tested substances were performed at 0, 24, 48, and 72 hours.

Thus, cells were divided into experimental Groups:

Group I (Control): Broth medium

Group II: Aqueous ozone at 2 µg/mL

Group III: Aqueous ozone at 5 µg/mL

Group IV: Aqueous ozone at 8 µg/mL

Each well of the plate was filled with 5 log^2^ cells/well. The experimental groups were comprised of 8 wells each. The ozonized PBS was applied into the cell culture of each group after 24 hours and remained in contact with the cells for 5 minutes. Then, it was removed and replaced by the medium broth. The MTT test was applied at 0, 24, 48, and 72 hours. The results were measured by an ELISA spectrophotometer with a 562 nm wavelength.

The data was recorded and submitted to statistical analysis using a two-way repeated measures analysis of variance with the Bonferroni *post hoc* test in case of significance to detect differences among the groups.

Data related to the cell count presented normality and homogeneity. Therefore, we opted to present the mean and standard deviation to represent these variables. As the samples were submitted to different ozone concentrations, and the cell viability was assessed at different time periods, we performed a two-way repeated measures analysis of variance. For all analyses, the level of significance was set at 5%. The analysis was performed using statistical software (MedCalc 12.1.4.0, MedCalc software bvba, Ostend, Belgium).

## RESULTS

### Microbiological assay

Statistical analyses provided comparison between control and experimental groups of the same bacterium.

The results revealed a decrease from 7.60 x 10^5^ CFU/mL in Group I to no CFU count of *Enterococcus faecalis* in Groups II, III, and IV. *Pseudomonas aeruginosa* was detected in Groups I, II, and III and it was not detected in Group IV, thus Group II and III showed statistical significance to Group I (p<0.05). Group IV was effective with no CFU count, statistically different from Groups I, II and III. *Staphylococcus aureus* showed a decrease from 6.80x10^5^ in Group I to 3.20x10^4^ in Group II. This reduction was statistically significant (p<0.05). In Groups III and IV, the CFU count was not detectable. Group IV was the most effective, with no CFU count (Table 1).

**Table 1:** Counts of colony forming units of *Enterococcus faecalis*, *Pseudomonas aeruginosa*, and *Staphylococcus aureus* from root canals treated with different methods

	*Enterococcus faecalis*	*Pseudomonas aeruginosa*	*Staphylococcus aureus*
	CFU/ml x 105	CFU/ml x 10^4^	CFU/ml x 10^5^
	Median (Interquartile range)		
Group I (Control)	7.60^A^ (1.04 – 10.8)	7.93^A^ (5.83 – 7.70)	6.80^A^ (5.27 – 9.33)
Group II	0.00^B^ (0.00 – 0.00)	1.44^B^ (0.68 – 6.30)	0.00^B^ (0.00 – 0.32)
Group III	0.00^B^ (0.00 – 0.00)	1.20^B^ (0.65 – 8.88)	0.00^C^ (0.00 – 0.00)
Group IV	0.00^B^ (0.00 – 0.00)	0.00^C^ (0.00 – 0.00)	0.00^C^ (0.00 – 0.00)

CFU=colony forming units

Different upper letters indicate statistically significant differences among the groups considering the same bacteria species (p<0.05 by Kruskal-Wallis test)

### Cytotoxicity assay

The results are expressed in Table 2. The Group I (control) was considered to be 100%. The results revealed that Groups III and IV provided the greatest decrease in cell viability at 0 hour, from 100% to 77.3% and 68.6%, respectively, which was statistically different from Group I (p<0.05) and different from Group II. After 24 hours, the cell viability increased in Groups II, III, and IV from 98.2%, 77.3%, and 68.6% at 0 hours to 103.2%, 104.3%, and 112.9%, respectively. No statistical significance was found between Groups III and IV, but there was a difference between Groups I and II. After 48 and 72 hours, the cell viability continued to increase, but this difference was not significant (p<0.05). Groups III and IV provided the greatest decrease in cell viability at 0 hour but the highest increase after 72 hours, with statistical differences among the groups (p<0.05).

**Table 2:** Viability of cells submitted to different ozone concentrations after different periods of time

Time	Ozone 2 µg/mL	Ozone 5 µg/mL	Ozone 8 µg/mL
	% of viability of the cells *		
	Mean (Standard deviation)		
0 h	98.2 (9.6)^A,a^	77.3 (14.8)^A,b^	68.6 (17.2)^A,b^
24 h	103.2 (15.1)^A,a^	104.3 (18.7)^B,a^	112.9 (18.1)^B,a^
48 h	105.5 (25.1)^A,a^	105.3 (28.8)^B,a^	115.6 (21.9)^B,a^
72 h	105.8 (16.2)^A,a^	107.5 (14.4)^B,a^	121.2 (24.1)^B,a^

* Compared with the viability of the cells in the control group, which was considered to be 100%. Different upper case letters indicate statistically significant differences among the different periods considering the same ozone concentration (p<0.05). Different lower case letters indicate significant differences among different ozone concentrations considering the same period (p<0.05)

## DISCUSSION

The scientific literature has already proven that traditional Endodontic therapy is not able to promote the root canal system’s sterilization^23^ ^-^ ^25^. Sodium hypochlorite is the most employed solution worldwide due to its extremely potent antimicrobial activity^22^ ^-^ ^25^ and tissue dissolution^21^. However, it has its deficiencies, such as a lack of biocompatibility^5^ ^,^ ^9^.

Ozone has been proposed as an alternative for Endodontic treatment due to its potent antimicrobial activity^2^ ^,^ ^6^ ^,^ ^7^ ^,^ ^10^ ^,^ ^12^ ^-^ ^18^ ^,^ ^20^ as well as its low cytotoxicity within cells, which is the opposite from the highest concentration of sodium hypochlorite, being remarkably cytotoxic^22^.

In parallel with cytotoxicity assays, ozone’s antimicrobial activity has already been tested^17^. However, the concentration of ozonated water in the present study was higher than that proposed by the study referred to^17^. Both studies, Nagayoshi, et at.^17^ (2004) and the present one verified the ozone’s cytotoxicity, but only the present assay evaluated three different concentrations.

The methodological model employed in this study evaluated a single concentration of aqueous ozone and ozone gas in an antimicrobial assay. A prior study provided the support to justify this conduct^20^. This study evaluated three different concentrations of aqueous ozone over *Enterococcus faecalis, Pseudomonas aeruginosa*, and *Staphylococcus aureus*, though in planktonic form. Despite the methodological limitations of this previous study, aqueous ozone in a concentration of 8 µg/mL was the most effective. Therefore, this information was transferred to the present study.

The present microbiological assay confirmed the effectiveness of the ozonated water against the tested bacteria. The tested microrganisms showed sensitivity to all the applied protocols. *Pseudomonas aeruginosa* was the most resistant but with a significant reduction from the Control (Group I). This data was in accordance with a prior study^12^ which evaluated the effectiveness of ozone against microrganisms organized in biofilm mono-species. The ozone group provided a decrease in CFU count but it was not able to completely eliminate *Psedomonas aeruginosa*. The present study provided no CFU count in Group IV, employing ozonated water as complement to Endodontic protocol.

Such a model confirmed the deficiency of the standard Endodontic protocols (Group II) for *Pseudomonas aeruginosa* and *Staphylococcus aureus* and the importance of a complementary therapy to improve root canal system decontamination (Group IV). Such data confirms the results achieved by different studies^6^ ^,^ ^12^ ^,^ ^16^ ^,^ ^18^ ^,^ ^20^. Rôças and Siqueira^23^ (2011) studied the complexity of the Endodontic infection and related it to the necessity of a complementary therapy to optimize the decontamination stage and eliminate the most resistant bacteria located in the root canal system. Thus, ozone was highlighted as a promising tool to fulfill this requirement specifically ozonated water which provided no CFU count for any of the tested microorganisms.

Aqueous ozone retains only 20–25% of the delivered ozone^3^. This statement was confirmed in a prior study^20^. Thus, to reach the final concentration of 8 µg/mL, the ozone device was calibrated to produce 40 µg/mL of ozone. According to the conclusion of this previous study^20^, this concentration was adopted as a standard for the present assay. The methodological chronology employed first evaluated the antimicrobial efficacy of ozone gas at 40 µg/mL and the same concentration was applied to aqueous ozone to reach a final concentration of 8 µg/mL. The results provided by microbiological assay encouraged the cytotoxicity analyses over the gingival fibroblasts.

A better comprehension of ozone’s action mechanism improves the correlation between the results of this study and their clinical appliance. As a variation of oxygen, one of ozone’s greatest features is its oxidative power^2^ ^,^ ^4^ ^,^ ^19^. The oxidative stress, which is caused by its oxidative power, is responsible for the reactive oxygen species (ROS) which are converted into free radicals such as singlet oxygen, hydrogen peroxide, and superoxide anions^2^ ^,^ ^8^ ^,^ ^19^.

Due to the production of free radicals, ozone has a remarkable antimicrobial effect. Singlet oxygen, hydrogen peroxide, and superoxide anions oxidate the polyunsaturated fatty acid of the bacterial cell wall membrane provoking the disruption of the bacterial membrane^2^ ^,^ ^10^ ^,^ ^13^ ^,^ ^15^ ^,^ ^19^.

On the other hand, when in contact with cells, ozone delivers full biochemical reactions, and the mitochondrion is the target^1^ ^,^ ^2^. Such a concept justified the choice to employ the MTT methodology in the present study to evaluate the mitochondrial activity^11^ ^,^ ^17^. Previous literature has shown the effects of ozone to produce oxidative stress^1^ ^,^ ^2^ ^,^ ^1^ ^,^ ^4^ ^,^ ^8^, which *in vitro* reflects a immediate decrease in cell viability^1^ ^,^ ^4^ confirmed by the cytotoxicity assay, decreasing cell viability from 100% to 98.2%, 77.3%, and 68.6% in Groups II, III, and IV, respectively. Free radicals are released to stimulate mitochondrion to produce adenosine triphosphate (ATP). Physiologically, this event represents the cells’ metabolic improvement^1^ and will improve cell viability^17^, which has been confirmed by the present study over the following experimental periods of 24, 48, and 72 hours. Groups III and IV provided the greatest increase in cell viability with no significance between them, but with a significant difference from Group II. These results are in accordance with studies that evaluated the cytotoxicity^11^ ^,^ ^17^ and biocompatibility^8^ of ozone.

Several studies have treated ozone isolated from other chemical substances^6^ ^,^ ^7^ ^,^ ^10^ ^,^ ^12^ ^-^ ^18^ ^,^ ^20^. The aim of this study was to evaluate the efficacy of ozone therapy but not as a new chemical solution that would substitute any component of the protocol. Thus, in this sense, these results confirm Lynch’s statement that ozone is most effective after traditional cleaning, shaping, and irrigation has been completed^15^.

Pure oxygen is extremely important to improve the production of ozone^1^ ^,^ ^2^ ^,^ ^19^, thus its clinical application. The ozone device employed in this study (Philozon, Balneario Camburiú, SC, Brazil) is indicated to the medical field, but adapted for Odontology. Clinical application in both areas, medicine and Odontology are similar when they cope with ozone as a topical agent. A syringe is connected to the ozone generator and filled with gas or connected to a glass flask to bubble ozone and produce aqueous ozone. Both forms, gas and aqueous are perfectly suitable to apply to clinical sites.

Commercially there are few ozone devices specific for dental application which use the oxygen as feedstock. Therefore the adaptation from medical to dental application must be perfectly suitable due to the simplicity of the technique.

Unfortunatelly, there is a lack of consistent studies to support the clinical application of ozonetherapy due to the lack of clinical trials. The complexity of the Endodontic infection associated to anatomical variations require a complement to Endodontic therapy, as studied^23^. Thus several doubts surround the therapy. This study, under limitations, opens a door and confirms previously studies^6^ ^,^ ^7^ ^,^ ^10^ ^-^ ^18^ ^,^ ^20^ to purpose ozone as a complementary procedure to improve root canal decontamination as part of the chemicalmechanical preparation.

## CONCLUSION

According to the applied methodology and the limitations of this study, it was possible to conclude that in association with standard Endodontic protocol, ozone therapy, and specifically aqueous ozone, improved the decontamination of the root canal *ex vivo*. In parallel, ozone was toxic to the gingival fibroblast on first contact, but the cell viability was recovered by the end of the experiment. Thus, these findings suggest that ozone has the potential to take part in Endodontic treatment, but further studies, especially clinical trials, must be encouraged.
